# SARS-CoV-2 Infection (COVID-19) and Rhinologic Manifestation: Narrative Review

**DOI:** 10.3390/jpm12081234

**Published:** 2022-07-28

**Authors:** Seung Hoon Lee, Min Young Seo

**Affiliations:** 1 Division of Rhinology, Department of Otorhinolaryngology-Head and Neck Surgery, Korea University Ansan Hospital, Ansan 15355, Korea; shleeent@korea.ac.kr; 2 Division of Rhinology, Department of Otorhinolaryngology-Head and Neck Surgery, Korea University College of Medicine, Seoul 02841, Korea

**Keywords:** SARS-CoV-2, COVID-19, olfaction, chronic rhinosinusitis, allergic rhinitis, rhinology

## Abstract

Patients with severe pneumonia of unknown etiology presented in December 2019 in Wuhan, China. A novel coronavirus, severe acute respiratory syndrome coronavirus-2 (SARS-CoV-2), was isolated from the respiratory tracts of these patients. The World Health Organization (WHO) defined respiratory diseases due to SARS-CoV-2 infection as coronavirus disease 2019 (COVID-19). Many researchers have reported that the nasal cavity is an important initial route for SARS-CoV-2 infection and that the spike protein of this virus binds to angiotensin-converting enzyme 2 (ACE2) on epithelial cell surfaces. Therefore, COVID-19 is thought to significantly affect nasal symptoms and various rhinological diseases. In this review, we summarize the association between COVID-19 and various rhinological diseases, such as olfactory dysfunction, rhinosinusitis, and allergic rhinitis.

## 1. Introduction

Several patients with severe pneumonia of unknown etiology presented in December 2019 in Wuhan, China. A novel coronavirus, severe acute respiratory syndrome coronavirus-2 (SARS-CoV-2), was isolated from the respiratory tracts of these patients. The World Health Organization (WHO) defined respiratory diseases due to SARS-CoV-2 infection as coronavirus disease 2019 (COVID-19). The SARS-CoV-2 infection is different from other respiratory viral infections because it does not show any symptoms despite the high viral loads in several cases. Therefore, controlling COVID-19 in its initial stage of viral infection is very difficult. During the initial phase of the COVID-19 pandemic, systemic symptoms such as fever, chills, and fatigue were regarded as cardinal symptoms of COVID-19; lower respiratory symptoms such as dyspnea, cough, and sputum were also regarded as important symptoms of COVID-19 [1,2]. However, following the publication of several cases of olfactory disturbances (hyposmia and anosmia) [3,4,5], nasal symptoms were also regarded among the main symptoms of COVID-19, and the American Academy of Otolaryngology-Head and Neck Surgery (AAO-HNS) proposed that anosmia and hyposmia be added to the list of initial screening tools for COVID-19 in March 2020. Thereafter, the Centers for Disease Control (CDC) and WHO added these symptoms to the list of symptoms of COVID-19 in April and May 2020, respectively. In addition, many researchers have reported that the nasal cavity is an important initial route for SARS-CoV-2 infections and that the spike protein of this virus binds to angiotensin-converting enzyme 2 (ACE2) on the epithelial cell surfaces of sinonasal mucosa [6,7,8]. Therefore, COVID-19 is thought to significantly affect nasal symptoms. Moreover, various rhinological diseases including allergic rhinitis and rhinosinusitis might also be significantly associated with COVID-19. In this literature review, we summarize the association between COVID-19 and various rhinological diseases such as olfactory dysfunction, rhinosinusitis, and allergic rhinitis.

## 2. Review

### 2.1. Olfactory Dysfunction

Olfactory dysfunction is regarded as a cardinal symptom of COVID-19. Since the early stages of the pandemic, various centers such as the WHO, CDC, and AAO-HNS have used this symptom as part of the initial screening for COVID-19 [9,10]. Therefore, numerous studies have been conducted on olfactory dysfunction in patients with COVID-19. A recently published article reported that in patients with long-lasting/relapsing olfactory dysfunction after a COVID-19 infection, SARS-CoV-2 RNA was detected in cytological samples from olfactory mucosa but not in nasopharyngeal samples [11]. Therefore, researchers determined that SARS-CoV-2 is persistent in the olfactory epithelium of COVID-19 patients with olfactory dysfunction, and that the olfactory dysfunction is linked to inflammation caused by persistent SARS-CoV-2 infection [11]. In addition, direct damage to the olfactory epithelium, followed by retrograde neuro-invasion of SARS-CoV-2 through the olfactory route, might also affect the olfactory function in COVID-19 patients [11].

The initial prevalence of olfactory dysfunction in hospitalized COVID-19 patients in Wuhan, China, was approximately 13.8% [12]. A study reporting this prevalence shows considerable variability according to researchers and diagnostic tools. A recently published meta-analysis of 27,492 patients reported that the overall prevalence of olfactory dysfunction in COVID-19 patients was 47.85% (95% CI: 41.20–54.50) [11]. The researchers also reported that the prevalence showed variability according to geographical differences: 54.40% in Europe, 51.11% in North America, 31.39% in Asia, and 10.71% in Australia [11]. However, these results include both subjective and objective olfactory disorders, which were assessed using questionnaires and psychophysical tests. When the results were separated according to screening tool (subjective questionnaires and objective psychophysical tests), the prevalence rates were 44.53% and 72.10%, respectively [11]. We also found another article about the prevalence of olfactory dysfunction that compares the subjectivity of olfaction. The authors conducted an evaluation of 2581 COVID-19 patients and reported that the prevalence of subjective olfactory dysfunction was 85.9% in mild, 4.5% in moderate, and 6.9% in severe COVID-19 patients. However, objective olfactory dysfunction was observed in 54.7% of mild and 36.6% of patients with moderate-to-severe COVID-19 [13]. Therefore, we also found that olfactory dysfunction is more prevalent in mild forms of COVID-19. When we consider the inconsistency between subjective and objective olfactory dysfunction, olfactory function in COVID-19 patients should not be assessed solely based on the patient’s statements and questionnaires.

The improvement rate of olfactory dysfunction in patients with COVID-19 is relatively higher than that of other symptoms. Regarding subjective olfactory function improvement, about 84.2% of patients stated that their olfactory function was ‘very good’ or ‘good’ 4 weeks after their symptom onset [14]. In addition, only approximately 10% to 25% of patients reported no improvement in their olfactory function. Approximately 48.7% of patients reported complete resolution of olfaction at 4 weeks after symptom onset [15,16,17,18]. Two months after symptom onset, only 24.1% of patients reported no improvement in their olfactory function [13]. Regarding objective olfactory function improvement, only 15.3% and 4.7% of patients did not show improvement at 2 and 6 months, respectively [13]. The authors also published an article about olfactory recovery in COVID-19 infection; 92.1% of patients stated that their olfaction was normalized 2 months after symptom onset. However, only 52.6% of patients were confirmed to have a normosmic status according to a psychophysical test such as the Cross-Cultural Smell Identification Test (CC-SIT) [19]. Considering these results, we also found that subjective olfactory function assessment using self-reporting questionnaires overestimates the degree of recovery. Therefore, an objective psychophysical test should be conducted to assess olfaction recovery.

To date, there has been no definitive treatment for COVID-19-associated olfactory dysfunction. Therefore, many physicians perform various therapeutic modalities, including olfactory training with and without systemic or topical corticosteroid supplementation. Olfactory training is the most validated therapeutic modality for olfactory dysfunction, developed by Hummel et al. [20] using the four odorants according to Henning’s odor prism: eucalyptus (resinous), clove (spicy), lemon (fruity), and rose (flowery). Repetitive exposure to odorants changes the olfactory epithelium, olfactory bulb, and even higher levels of olfactory perception such as neuroplasticity [20]. Functional connectivity, such as olfactory, somatosensory, and integrative networks, increases after olfactory training [21]. Olfactory training is a widely used therapeutic modality especially in patients with post-infectious olfactory dysfunction. Post-infectious olfactory dysfunction occasionally occurs because of upper airway viral infection, in which olfactory impairment persists even after the improvement in other respiratory symptoms [19]. Previously, the authors reported that COVID-19-associated olfactory dysfunction was regarded as a quantitative disorder (hyposmia or anosmia) with a sensorineural cause. These clinical characteristics are similar to those of post-infectious olfactory dysfunction. Therefore, the authors regarded that olfactory training might be significantly effective in treating COVID-19-associated olfactory dysfunction, as do most physicians also looking at it from the same point of view [22].

Several studies have reported the effects of olfactory training in patients with COVID-19-associated olfactory dysfunction and that subjective and objective olfactory functions were significantly improved after olfactory training [23,24]. Denis et al. performed olfactory training with a mean visual stimulation duration of 4 weeks in 548 patients with COVID-19-associated olfactory dysfunction. They reported that the recovery rate was approximately 73.3% for the group of patients that trained for more than 4 weeks and 59% for the group that trained less than 4 weeks [23]. Altundag et al. performed modified olfactory training using 12 odorants during a 36-week period on 75 patients with COVID-19-associated parosmia, and reported a significant improvement in olfactory function in both treatment and non-treatment groups at the third, sixth, and ninth months. However, the degree of improvement was significantly higher in the treatment group than in the non-treatment group [24]. The authors also published an article about the effects of olfactory training in patients with persistent olfactory dysfunction for about 3 months. They found that approximately 70% of patients were normalized according to psychophysical tests (using CC-SIT), after two months of olfactory training using four common Korean odorants (pine, peppermint, cinnamon, and lemon) [19]. Moreover, according to the Clinical Olfaction Working Group, 89% of physicians fully or partly agreed on olfactory training for the treatment of COVID-19-associated olfactory dysfunction [25]. Therefore, olfactory training might be an effective therapeutic modality for COVID-19-associated olfactory dysfunction without having significant side effects.

Systemic or topical steroid supplementation is another therapeutic option for the treatment of olfactory dysfunction and has anti-inflammatory effects. However, there is a lack of evidence regarding the treatment of post-infectious olfactory dysfunction. Several studies have evaluated topical or systemic steroid supplementation and have shown considerable variability. Le Bon et al. performed a prospective case-control study and suggested that a combination of a short course of oral corticosteroids (32 mg methylprednisolone for 10 days) and olfactory training is safe and may be beneficial [26]. In addition, Kasiri et al. [27] reported that a combination of intranasal steroids and olfactory training significantly increased recovery rates. However, Saussez et al. reported that the objective olfactory function, using a threshold, discrimination, and identification (TDI) score, was significantly improved after treatment in all groups (group 1 (0.5 mg/kg/day methylprednisolone for 10 days with olfactory training, *n* = 59) vs. case 2 (2 puffs (100 μg) mometasone furoate once daily with olfactory training, *n* = 22) vs. control (olfactory training alone, n = 71)), with the highest degree of improvement observed in the systemic steroid supplement group after one month of treatment. However, at two months after treatment, this superiority did not remain: the degree of olfactory improvement in the other groups became too similar to that of the systemic steroid supplement group. [28] Therefore, they suggested that topical or systemic steroid supplementation is not favorable when considering the risk–benefit ratio, and Abdelalim et al. reported that a combination of intranasal steroids and olfactory training did not show superiority over olfactory training alone [29]. In addition, according to the Clinical Olfaction Working Group, 84% of physicians fully or partly disagreed with systemic steroids as a first-line treatment for COVID-19-associated olfactory dysfunction, and 95% of physicians fully or partly agreed that systemic steroids should not be considered as a standard treatment for patients with COVID-19-associated olfactory dysfunction [25]. Therefore, we do not recommend the use of systemic steroids until clear evidence is presented; we only recommend the use of topical steroids in cases of clinical necessity, such as concomitant nasal symptoms, nasal obstruction, rhinorrhea, sneezing, and itching.

### 2.2. Rhinosinusitis

As mentioned above, the spike protein of SARS-CoV-2 binds to ACE2 on epithelial cell surfaces for primary invasion. Therefore, alterations in ACE2 expression may affect COVID-19 infection. According to a recently published article, ACE2 expression in the sinonasal mucosa is lower in eosinophilic chronic rhinosinusitis (CRS) patients with type-2 inflammation than in patients with non-eosinophilic CRS or control subjects [30]. In addition, ACE2 regulation is positively correlated with proinflammatory cytokines such as tumor necrosis factor (TNF) and interleukin (IL)-1β in CRS patients, and negatively correlated with eotaxin-3, which chemokines related with eosinophil [30]. Another study reported that the expression of ACE2 in the sinonasal mucosa is influenced by the CRS endotypes in patients with nasal polyps. In nasal epithelial cells, type 1 inflammation increases ACE2 expression, while type 2 inflammation decreases it [31]. Therefore, CRS type might be related to SARS-CoV-2 transmission, and an understanding of these findings might contribute to the prevention and control of COVID-19 infection [30,31].

According to the European position paper on rhinosinusitis and nasal polyps 2020 (EPOS 2020), coronavirus is the most common virus isolated from acute rhinosinusitis and acute exacerbating chronic rhinosinusitis [32]. SARS-CoV-2 is a member of the *Coronaviridae* family, so it causes similar clinical symptoms to other members of the *Coronaviridae* family. A recently published clinical study reported that clinical diagnosis of acute rhinosinusitis according to EPOS 2020 could be confirmed in approximately 45% of COVID-19 patients. Furthermore, headache is significantly associated with acute rhinosinusitis symptoms in COVID-19 patients, and nasal symptoms were more prevalent in the COVID-19 patients with headache than headache-free COVID-19 patients [33].

According to the clinical data of hospitalized COVID-19 patients in Wuhan, China, the prevalence of CRS in COVID-19 patients was 6.1%, which was not significantly different from that of the general Chinese population (8%) [34]. However, CRS patients with COVID-19 had a significantly higher frequency of concomitant asthma than CRS patients without COVID-19 (6.9% vs. 6.2%, respectively) [34]. In addition, CRS patients with COVID-19 tended to more frequently suffer from fever than CRS patients without COVID-19 without statistical significance (87.5% vs. 78%, respectively) [34]. Another clinical study with a sample of over 12,000 CRS patients, also reported that the CRS patients had a significantly higher frequency of COVID-19 testing than the control population (27.5% vs. 15.3%, respectively). However, the prevalence of COVID-19 was not significantly different between CRS and non-CRS patients (both 1.4%) [35]. In addition, researchers reported that CRS comorbidity was not associated with COVID-19 severity [34,35]. However, another study reported that the median duration of viral clearance was 23 days in COVID-19 patients without CRS and 48.5 days in COVID-19 patients with CRS. the authors concluded that CRS was independently associated with prolonged viral shedding in COVID-19 patients [35]. Therefore, this finding might have clinical implications for quarantine duration owing to the increased risk of pandemic spread [36].

To date, there have been many reports on the association between acute invasive fungal rhinosinusitis and COVID-19 infection [37,38,39,40]. Abdelsamie et al. presented a cross-sectional cohort study of 22 adult COVID-19 patients with concomitantly confirmed acute invasive fungal rhinosinusitis. They reported that all patients had diabetes mellitus, and 77.3% of patients were treated with systemic steroid supplementation. Among these 22 patients, 20 patients were treated with intravenous liposomal amphotericin B therapy, and surgical management was performed in 18 patients. According to the pathological results, mucormycosis was confirmed in 19 patients (86.4%) and aspergillus in only 3 patients (13.6%). The treatment outcome included total improvement in 10 patients (45.5%), intracranial extension in 10 patents (45.5%), and 6 patients died from the disease (the mortality rate was 27.3%) [37]. Dilek et al. systematically reviewed COVID-19-associated mucormycosis in 100 patients. They reported that the highest prevalence is in India (*n* = 68), and 76% were men. The most frequently involved sites were the rhino-orbital complex (*n* = 50), sinonasal (*n* = 17), and rhino-orbito cerebral (*n* = 15) sites. The overall survival rate was approximately 66.7% [38]. According to these articles, the most common risk factors have been corticosteroid use and diabetes mellitus [37,38]. Immune suppression observed in COVID-19 patients was attributed to a decrease in CD4+ and CD8+ T cells [41]. Therefore, COVID-19 patients are more vulnerable to fungal infections. In cases of acute invasive fungal rhinosinusitis, the treatment of choice is management of underlying disease and aggressive surgical debridement. Elmokadem et al. recently published an article about post-operative imaging outcomes in COVID-19 associated acute invasive fungal rhinosinusitis. They reported that 72% of patients showed rapid progression, newly developed intracranial extension, residual/recurrent osteonecrosis, or post-operative facial defects. In addition, 20% of patients showed residual infection, and conservative management with antifungal therapy was performed [42].

### 2.3. Allergic Rhinitis

To the best of our knowledge, there have been no studies on the direct effect of SARS-CoV-2 infection on allergic rhinitis. However, according to two studies, using a COVID-19 protective facial mask during pollen season may reduce the symptoms of allergic rhinitis [43,44]. Dubini et al. reported that ragweed-allergy -related nasal symptoms (sneezing, rhinorrhea, nasal obstruction, and nasal itching) significantly improved in the 2020 ragweed season compared with 2019 [43]. However, ocular symptoms (watering eyes, swollen eyes, eye itching, and tired or sore eyes) were not significantly different between the 2020 and 2019 ragweed seasons [43]. In addition, Liccardi et al. reported that spring seasonal-allergy-related (Parietaria, grasses, and Olea europaea) nasal symptoms (sneezing, rhinorrhea, nasal obstruction, and nasal pruritus) significantly improved in April 2020 compared with April 2019 [43]. However, ocular symptoms (ocular pruritus and tearing) were modest or not significantly different between April 2020 and April 2019 [43]. Therefore, we determined that using a facial mask significantly improves nasal allergic symptoms during both spring and autumn seasons. However, it does not reduce the ocular allergy symptoms.

We found a statement regarding allergic immunotherapy for allergic rhinitis in COVID-19 patients. In patients with COVID-19, lymphopenia affects the T-cell response; severe inflammatory responses, including cytokine storm in severe patients; Th1-Th2 responses; and significant antibody levels increases. Therefore, the response to immunotherapy may be significantly different in COVID-19 patients. According to the Allergic Rhinitis and Its Impact on Asthma-European Academy of Allergy and Clinical Immunology (ARIA-EAACI) statement, in non-infected individuals or patients who have recovered from COVID-19, interrupting subcutaneous immunotherapy (SCIT) is not advised, but expanding injection intervals in the continuation phase may be beneficial. In addition, the interruption of sublingual immunotherapy (SLIT) is not advised and can be performed at home. In COVID-19 patients, interruption of SCIT and SLIT is recommended [45].

## 3. Conclusions

We summarized the literature regarding the association between COVID-19 and various rhinological diseases such as olfactory dysfunction, rhinosinusitis, and allergic rhinitis in this article. Because the nasal cavity is an important initial route for SARS-CoV-2 infection, it significantly affects nasal symptoms and various rhinological diseases. Olfactory dysfunction is the most common symptom of COVID-19-associated nasal symptoms. A recently published meta-analysis reported that the overall prevalence of olfactory dysfunction in COVID-19 patients is 47.85%. However, when only using screening tools such as objective psychophysical tests, the prevalence rate increases up to 72.10%. The natural improvement rate of olfactory dysfunction in COVID-19 patients is approximately 84.2%, and about 50% of patents were normalized 4 weeks after symptom onset. In addition, when only considering the objective olfactory function assessment, about 85% and 95% of patients were improved at 2 and 6 months, respectively. Therefore, we concluded that the improvement rate is relatively higher than that of other causes. However, authors have found that subjective assessment of olfactory function overestimates the degree of recovery. Therefore, we recommended that an objective psychophysical test should be conducted to assess olfaction recovery. When considering the treatment modality for olfactory dysfunction in COVID-19 patients, olfactory training is the only validated and recommended treatment regarding risk and benefit. Additionally, routine systemic steroid supplement should not be considered as a standard treatment, and the use of topical steroids is only recommended in cases of concomitant nasal symptoms.

Alterations in ACE2 expression in the sinonasal mucosa may affect COVID-19 infection. To the best of our knowledge, ACE2 expression in the sinonasal mucosa is influenced by inflammatory endotypes of CRS. Expression is lower in eosinophilic CRS with type 2 inflammation and higher in non-eosinophilic CRS with type 1 inflammation. Therefore, non-eosinophilic CRS with type 1 inflammation might increase SARS-CoV-2 infection through sinonasal mucosa because of ACE2 upregulation. Immune suppression and decreased CD4+ and CD8+ T cells in COVID-19 patients make them more vulnerable to fungal infections. Therefore, the association between acute invasive fungal rhinosinusitis and COVID-19 infection has been reported in numerous articles. In COVID-19 patients, the overall mortality rate is about 30%, and major concomitant medical problems are diabetes mellitus and systemic steroid supplementation.

COVID-19 significantly alters the immunological process and affects the planning and progression of allergic immunotherapy. According to the ARIA-EAACI statements, immunotherapy should be discontinued in COVID-19 patients.

In conclusion, physicians should be aware of the association between COVID-19 and various rhinological diseases and consider this when treating patients.

## Data Availability

Not applicable.
